# Socioeconomic inequalities in general and psychological health among adolescents: a cross-sectional study in senior high schools in Greece

**DOI:** 10.1186/1475-9276-9-3

**Published:** 2010-01-23

**Authors:** Konstantina Magklara, Petros Skapinakis, Dimitrios Niakas, Stefanos Bellos, Anastasia Zissi, Stylianos Stylianidis, Venetsanos Mavreas

**Affiliations:** 1 Department of Psychiatry, University of Ioannina, School of Medicine, Greece; 2 School of Social Sciences, Hellenic Open University, Patras, Greece; 3 Department of Sociology, University of the Aegean, Mytilene, Greece; 4 Department of Psychology, Panteion University of Social and Political Sciences, Athens, Greece

## Abstract

**Background:**

Socioeconomic health inequalities in adolescence are not consistently reported. This may be due to the measurement of self-reported general health, which probably fails to fully capture the psychological dimension of health, and the reliance on traditional socio-economic indicators, such as parental education or occupational status. The present study aimed at investigating this issue using simple questions to assess both the physical and psychological dimension of health and a broader set of socioeconomic indicators than previously used.

**Methods:**

This was a cross-sectional survey of 5614 adolescents aged 16-18 years-old from 25 senior high schools in Greece. Self-reported general and psychological health were both measured by means of a simple Likert-type question. We assessed the following socio-economic variables: parents' education, parents' employment status, a subjective assessment of the financial difficulties experienced by the family and adolescents' own academic performance as a measure of the personal social position in the school setting.

**Results:**

One out of ten (10%) and one out of three (32%) adolescents did not enjoy good general and psychological health respectively. For both health variables robust associations were found in adolescents who reported more financial difficulties in the family and had worse academic performance. The latter was associated with psychological health in a more linear way. Father's unemployment showed a non-significant trend for an association with worse psychological health in girls only.

**Conclusions:**

Socioeconomic inequalities exist in this period of life but are more easily demonstrated with more subjective socioeconomic indicators, especially for the psychological dimension of health.

## Background

Almost three decades following the publication of the Black Report [1] socioeconomic health inequalities are still a topic of considerable scientific interest. While social gradients in health are well established for both children [2] and adults [3], the relevant evidence for adolescent health is much less consistent. Although some studies have reported significant inequalities in adolescent health [4-10], other studies failed to confirm this [11-14]. The inconsistency of evidence led some researchers to argue that adolescence may be a period in the life cycle that is characterised by "social equalization in health" [15].

A number of methodological issues may help explaining these inconsistencies. First, measurement of socioeconomic status in adolescence is not straightforward. Parental socioeconomic characteristics, such as parental educational level, social/occupational class and family income, are often used as measures of the socioeconomic status of adolescents. It is suggested however that adolescents may not be able to accurately report their parent's socioeconomic status [10]. In addition, research in adults has shown that inclusion of more subjective indicators (for example presence of financial difficulties in the household) may be more strongly associated with health status [16]. Similar findings have been reported in adolescent studies [7]. Some studies have also attempted at assessing adolescents' own socioeconomic status on the grounds of their own educational level or current occupation [17]. However, this is not possible in countries where most of the adolescents remain in schools until the age of 18 years. In these countries academic performance in school has been used as an alternative estimation of adolescent's own social position revealing significant associations with health status [18].

Second, concerning health outcomes, self-reported health is one of the most frequently used health indicators in studies about adolescent health. Self-reported health is usually measured by means of a single question asking respondents to rate their recent overall health status on a Likert-type scale from excellent to poor. It has been used in many different cultural contexts and the findings indicate that it is a powerful indicator of clinical outcome and mortality [19], which correlates well with other more complex health indexes [20]. It has been argued though that the question about overall health may fail to capture the psychological dimension of health, since it relates principally to physical health problems [21]. This is potentially very important as several studies have highlighted the significant burden of psychological problems in adolescence [22-26]. While the inclusion of a detailed examination of the mental health state of adolescents in general surveys may not be feasible, studies that have used simple ways to crudely assess the psychological component of health, alongside the general question on self-reported health, have reported significant socioeconomic inequalities in some instances [27].

The aim of the present paper was to investigate this issue by studying the association between socioeconomic status and both general and psychological health, assessed in a similar crude way, in a sample of Greek adolescents. We hypothesized that the association between socioeconomic status and health of the adolescents would be more evident in the psychological component of health compared to general health.

## Methods

### Description of the data set

The data reported in this paper were derived from the "Epirus School Project" [28] which is a cross-sectional survey carried out in selected upper secondary schools in Greece. The principal aim of the survey was to investigate the prevalence and associations of common mental disorders in late adolescence.

### Secondary Education in Greece

Secondary education in Greece is distinguished into lower secondary (grades 7-9; attendance is compulsory) and upper secondary (grades 10-12; attendance is not compulsory). Upper secondary schools are further distinguished into Senior High Schools (Lyceum) and Technical Vocational Schools. The majority of students (75%) attend Senior High Schools. In the "Epirus School Project" only Senior High Schools were selected, while Technical Vocational Schools will be included in a separate future survey. At the time of the design of the study approximately 75000 students attended 1193 Senior High Schools.

### Sampling of Schools and Pupils

Schools were selected according to the following rules: a) all Senior High Schools of the major cities in the north-western part of Greece (regions of Epirus and Aetoloakarnania) due to the proximity of this area to the University of Ioannina, b) all Senior High Schools in one randomly selected district of the Athens Metropolitan Area (the district of Kallithea was selected), c) all Senior High Schools of the island of Paros in the Aegean Sea (the island was conveniently selected). A total of 25 schools took part in the study. The median number of participants per school was 225 pupils ranging from 138 to 425. The main fieldwork took place between January 2007 and April 2008.

The "Epirus School Project" used a two-phase design [29]. In the current paper we used data collected during the first phase only. In phase 1 all students in the selected schools were invited to participate in the study. Participation in the study was voluntary. Consent for participation was actively obtained from both the students and their parents. The first phase took place in the classroom, where all consenting students (N = 5614: boys = 45%/girls = 55%) were administered a self-completed paper and pencil questionnaire that included: a) sociodemographic questions and b) the screening instrument. The response rate in this phase was 82%, with the main reason of non-response being absence.

### Assessment of self-reported health

We used two health indicators in the present study: self-reported general health and self-reported psychological health. Self-reported general health was measured by means of the common question, by which respondents are asked to rate their recent general health status. There were five possible responses: "excellent", "very good", "good", "fair" and "poor". Similarly, self-reported psychological health was measured by asking respondents to rate their recent psychological state. There were again five possible answers: "excellent", "very good", "good", "fair" and "poor". We should like to note that in the context of the present study we have also collected several other measures of psychological health including the detailed fully-structured psychiatric interview on the second phase (CIS-R) [30]. On a subset of the first phase consisting of 10 schools and 2363 participants we have also given the Strengths and Difficulties Questionnaire (SDQ) [31] and the 12-item General Health Questionnaire (GHQ-12) [32]. In the present paper we have used the simple rating of psychological health for two reasons: a) our main aim was to study the association between socio-economic status and general health and to compare this with a similar simple question that explicitly asks about the perceived level of psychological health and b) these two questions were asked in the whole sample of the first phase, allowing us to use the full data set (5614 pupils from 25 schools).

### Assessment of Socioeconomic Status (SES)

We used the following SES indicators:

#### a) Parental Education

We measured the socioeconomic characteristics of both parents, since evidence shows that maternal socioeconomic characteristics have an equally, or even more, significant impact on child and adolescent health [33]. Pupils were asked to report their parents' highest educational level attained.

#### b) Parental Employment

Regarding employment, we chose to use the variable "employment type", which discriminates/distinguishes not only between employment status (employed - unemployed), but also between the different sectors of employment (self-employed, private and public sector employees). More specifically "employment type" was divided into seven groups: "public sector employee"; "private sector employee"; "self-employed"; "pensioner"; "unemployed"; "housewife" (as regards the mother's employment type) or "inability to work" (as regards the father's employment type) and "else". In Greece the legislative framework relating to the combination of family life and career (i.e. maternity leave, leave for breast-feeding/child bearing/illness, child/family allowances etc.) differs significantly between public and private sector [34]. Greek social research employs this kind of typology which links to the institutions and the way public life is organized in Greece. As a result the distinction between self-employed, private and public sector employees appears as very important for the country, since it determines in a great extent the way families live and function. Moreover, it is expected that the information lost by failing to ask the exact occupation, could be substituted by the information carried by the variable of education [35].

#### c) Financial status

It has been suggested that directly questioning adolescents about their family's income can be unreliable [36]. Therefore we asked the adolescents to express their subjective view on any financial difficulties their family might experience recently. The specific question asked was: "How do you think that your family is doing financially?" The possible answers included: "My family experiences no financial difficulties", "My family experiences very few financial difficulties", "My family experiences some financial difficulties" and "My family experiences a lot of financial difficulties". This type of question takes into account adolescents' subjective view of their family's economic position.

#### d) Personal social position of adolescents in the school context

In Greece, where typical 16-18 years-old adolescents have not yet entered the labour market, neither have they completed their education, own educational level or occupation cannot be used as a measure of personal social position. Academic performance in school has been often used as a measure of the social position of the pupils in school [18]. We therefore included academic performance in our SES indicators by asking the participants to rate their school performance (based on their recent marks) in a Likert scale with four choices ("excellent"; "very good"; "good"; "fair").

### Other Variables

Questions about several sociodemographic variables were also included in the self-reported questionnaire (own age, parent's age, gender, parent's marital status, number of brothers and sisters).

### Data analysis

The analyses were all conducted using the statistical software package STATA 9.0. Self-reported general health and self-reported psychological health were treated as dichotomous variables, which can take the following values: "poor health", which represents the sum of the values "fair" and "poor" of the initial variables self-reported general and psychological health, and "good health", which represents the sum of the values "good", "very good" and "excellent".

The association between health measures and sociodemographic and socioeconomic variables were investigated in a series of logistic regression models. To take into account the potential clustering of our data (since adolescents were nested into 25 schools) we carried out a two-level logistic model (level 1: individuals, level 2: schools) in STATA using the xtlogit command [37]. The xtlogit command employs Gauss-Hermite quadrature to evaluate and maximize the marginal log likelihood. We used the adaptive quadrature method ("aghermite" option in xtlogit command) as described by Rabe-Hesketh & Skrondal (2008). Initially, crude odds ratios were calculated. Then we adjusted for the sociodemographic and socioeconomic variables. Analyses were conducted separately for general and psychological health.

In order to compare gender differences in proportions of self-reported general and psychological health we used Pearson's chi square. The correlation between the Likert-type question about self-reported psychological health and the instruments of GHQ-12 and SDQ (emotional sub-scale) was calculated using Spreaman's rho.

## Results

Baseline socio-demographic and socioeconomic characteristics of the sample are shown in Table 1. Most students (89%) reported that their parents were married, while divorce or separation was reported by 7% of the pupils. Forty percent of the fathers and thirty five percent of the mothers had a degree from a university or technological institute. Almost one third of the fathers were employed in the public sector (33%), while a similar proportion (35.5%) were self-employed. Approximately one third of the mothers (35%) were looking after the house, while another third were working in the public sector. One in seven pupils reported that their family had at least some financial difficulties, while one in five rated their school performance as "fair".

**Table 1:** Basic description of the sample of 5614 adolescents in Greece.

Gender		Number of brothers/sisters	
Male	2530 (45%)	None	446 (8%)
Female	3084 (55%)	One	3087 (55%)
		Two	1313 (24%)
		Three or more	722 (13%)
**Age**		**Parents' Age**	
16	2265 (41%)	Father's age	47.97 (5.14)
17	1869 (33%)	Mother's age	42.60 (4.70)
18	1440 (26%)		

**Grade**		**Parent's Marital Status**	
10th grade	2281 (41%)	Married	5012 (89%)
11th grade	1772 (31%)	Divorced/Separated	394 (7%)
12th grade	1561 (28%)	Widow	159 (3%)
		Other/Missing	49 (1%)

**Father's Employment Type**		**Mother's Employment Type**	
Employed - Public Sector	1828 (33%)	Employed - Public Sector	1679 (30%)
Employed - Private Sector	1183 (22%)	Employed - Private Sector	1056 (19%)
Self-employed	1949 (35.5%)	Self-employed	741 (13%)
Retired	290 (5%)	Looks after House	1720 (31%)
Unemployed	42 (0.5%)	Unemployed	234 (4%)
Other/Missing	224 (4%)	Other/Missing	184 (3%)

**Father's Educational Level**		**Mother's Educational Level**	
Primary	791 (14%)	Primary	743 (13%)
Secondary Basic	849 (15.5%)	Secondary Basic	784 (14%)
Secondary Complete	1589 (29%)	Secondary Complete	2086 (37.5%)
Technological degree	738 (13.5%)	Technological degree	584 (10.5%)
University degree	1562 (28%)	University degree	1385 (25%)

**Financial Difficulties**		**School Performance**	
None	1776 (32%)	Excellent	510 (9%)
Very few	3028 (54%)	Very Good	1898 (34%)
Some	675 (12%)	Good	2135 (38%)
A lot	118 (2%)	Fair	1047 (19%)

Table 2 shows the level of self-reported general and psychological health of the adolescents. Among the 5614 adolescents 10% (girls: 13%, boys: 7%, p < 0.001) reported fair or poor general health, whereas the respective prevalence for fair or poor psychological health was 32% (girls: 42%, boys: 20%, p < 0.001). General and psychological health were significantly correlated with a spearman's rho of 0.41.

**Table 2:** Self-reported psychological health and self-reported general health in 5614 16-18 years-old adolescents, by sex.

	Excellent	Very Good	Good	Fair	Poor
**General Health**					
Boys	982 (38.84%)	972 (38.45%)	403 (15.94%)	125 (4.94%)	46 (1.82%)

Girls	607 (19.73%)	1245 (40.47%)	821 (26.69%)	351 (11.41%)	52 (1.69%)

**Psychological health**					
Boys	539 (21.34%)	801 (31.71%)	690 (27.32%)	355 (14.05%)	141 (5.58%)

Girls	271 (8.80%)	626 (20.33%)	903 (29.33%)	962 (31.24%)	317 (10.30%)

Crude and adjusted odds ratios for both self-reported psychological and general health are shown in Table 3. Among the sociodemographic factors studied, female gender, older age (attendance of higher grade) and parental divorce were all significantly associated with worse psychological health. With the exception of parental divorce, the same associations were reported for self-reported general health but the gender effect was weaker.

**Table 3:** Logistic regression analyses for self-reported psychological health and self-reported general health.

Variable	Self-reported Psychological Health		Self-reported General Health	
	**Crude ratios OR (95% CI)**	**Adjusted ratios OR (95% CI)**	**Crude ratios OR (95% CI)**	**Adjusted ratios OR (95% CI)**

**Gender**	2.91 (2.57-3.28)	3.06 (2.69-3.47)	2.08 (1.72 - 2.51)	2.12 (1.75-2.57)

**Grade**				
10^th^	1.00	1.00	1.00	1.00
11^th^	1.24 (1.09-1.43)	1.22 (1.06-1.42)	1.45 (1.18-1.80)	1.43 (1.15-1.77)
12^th^	1.65 (1.44-1.90)	1.61 (1.39-1.87)	1.64 (1.32-2.03)	1.57 (1.26-1.96)

**Parent's Marital Status**				
Married	1.00	1.00	1.00	1.00
Divorced/Separated	1.53 (1.24-1.89)	1.29 (1.03-1.63)	1.50 (1.10-2.02)	1.23 (0.89-1.69)
Widow	1.34 (0.96-1.85)	1.09 (0.69-1.74)	1.43 (0.89-2.28)	1.08 (0.56-2.05)
Other	2.07 (1.16-3.71)	1.90 (1.01-3.60)	5.00 (2.70-9.23)	4.39 (2.26-8.51)

**Father's age**	1.00 (1.00-1.01)	1.00 (0.99-1.02)	0.99 (0.99-1.00)	1.00 (0.98-1.02)

**Mother's age**	1.00 (1.00-1.01)	1.00 (0.98-1.01)	0.99 (0.98-1.00)	0.99 (0.97-1.01)

**Father's Employment Type**				
Public sector employee	1.00	1.00	1.00	1.00
Private sector employee	1.09 (0.93-1.27)	1.02 (0.86-1.22)	0.99 (0.77-1.26)	0.89 (0.68-1.16)
Self-employed	1.07 (0.94-1.23)	1.03 (0.88-1.21)	1.09 (0.88-1.34)	0.94 (0.74-1.20)
Retired	1.14 (0.88-1.49)	1.07 (0.80-1.43)	1.20 (0.81-1.78)	1.15 (0.76-1.74)
Unemployed	2.34 (1.27-4.32)	1.28 (0.65-2.49)	1.86 (0.82-4.26)	0.91 (0.38-2.17)
Other	1.54 (1.16-2.05)	1.10 (0.79-1.52)	1.28 (0.83-1.97)	0.87 (0.54-1.40)

**Mother's Employment Type**				
Public sector employee	1.00	1.00	1.00	1.00
Private sector employee	1.14 (0.97-1.35)	1.01 (0.83-1.23)	1.19 (0.92-1.54)	1.10 (0.82-1.47)
Self-employed	0.97 (0.80-1.17)	0.91 (0.73-1.13)	1.32 (0.99-1.75)	1.32 (0.97-1.82)
Retired/Looks after house	1.08 (0.93-1.25)	1.04 (0.87-1.24)	1.18 (0.94-1.48)	1.22 (0.93-1.60)
Unemployed	1.46 (1.10-1.94)	1.12 (0.82-1.53)	1.33 (0.86-1.52)	1.09 (0.69-1.74)

**Father's Educational Level**				
Primary	1.00	1.00	1.00	1.00
Secondary Basic	0.73 (0.59-0.90)	0.81 (0.65-1.02)	0.84 (0.62-1.13)	0.98 (0.71-1.35)
Secondary Complete	0.74 (0.61-0.88)	0.93 (0.77-1.25)	0.72 (0.55-0.95)	0.92 (0.68-1.24)
Technological degree	0.79 (0.64-0.98)	0.98 (0.78-1.26)	0.87 (0.64-1.19)	1.08 (0.76-1.54)
University degree	0.77 (0.64-0.92)	1.16 (0.92-1.47)	0.73 (0.56-0.96)	1.06 (0.75-1.49)

**Mother's Educational Level**				
Primary	1.00	1.00	1.00	1.00
Secondary Basic	0.70 (0.56-0.86)	0.84 (0.67-1.06)	0.68 (0.49-0.93)	0.78 (0.56-1.08)
Secondary Complete	0.68 (0.57-0.81)	0.87 (0.71-1.07)	0.64 (0.49-0.82)	0.82 (0.61-1.09)
Technological degree	0.68 (0.54-0.85)	0.83 (0.63-1.09)	0.67 (0.48-0.95)	0.88 (0.59-1.30)
University degree	0.68 (0.56-0.82)	0.94 (0.73-1.21)	0.70 (0.34-0.92)	1.08 (0.75-1.55)

**Financial Difficulties**				
No	1.00	1.00	1.00	1.00
Very few	1.34 (1.17-1.53)	1.26 (1.10-1.45)	1.62 (1.30-2.01)	1.56 (1.25-1.96)
Some	2.57 (2.14-3.10)	2.31 (1.89-2.83)	2.67 (2.02-3.53)	2.46 (1.84-3.30)
A lot	4.66 (3.17-6.85)	4.01 (2.65-6.08)	5.68 (3.66-8.81)	4.73 (2.95-7.59)

**School Performance**				
Excellent	1.00	1.00	1.00	1.00
Very good/Good	1.33 (1.08-1.65)	1.40 (1.12-1.75)	1.04 (0.75-1.43)	1.01 (0.73-1.42)
Fair	2.01 (1.59-2.54)	2.22 (1.71-2.87)	1.87 (1.32-2.65)	1.67 (1.15-2.41)

Crude odds ratios and odds ratios after adjusting for all sociodemographic and socioeconomic variables* calculated for self-reported psychological health and self-reported general health in 5614 16-18 years-old adolescents in Greece.

* Odds ratios adjusted for all other variables of the table.

OR: Odds ratio

CI: Confidence Interval

In the unadjusted analysis, pupils who had a mother or father with higher than primary education were less likely to report poor psychological health, while regarding general health only mother's educational level consistently showed this association. However, adjustment for the various sociodemographic and socioeconomic variables reduced these associations which became non significant.

In the unadjusted analysis pupils with unemployed fathers were more likely to report poor psychological health and a similar but not significant trend was found for general health. Adjustment for the remaining socioeconomic variables and especially the presence of financial difficulties in the family weakened this association. In a separate analysis for boys and girls it became clear that the effect of father's unemployment was evident only in girls (crude odds ratio for poor psychological health: 3.63, p = 0.003) but further adjustment for the remaining variables resulted in a non significant odds ratio for poor psychological health in girls (odds ratio: 2.20, p = 0.08).

The presence of financial difficulties in the family was significantly associated with both worse psychological and general health showing additional evidence of a dose-response relationship (Table 3, columns 3 and 5). Similarly, the adolescent's school performance was linearly associated with worse psychological health, while in the case of general health this was only evident for those pupils who rated their performance as only fair.

## Discussion

The aim of our analyses was to investigate socioeconomic health inequalities in a sample of Greek adolescents. Since our aim was not the estimation of prevalence rates, we identified high-risk groups by using two simple questions, one about general health and one about psychological health. In our sample one out of ten adolescents did not enjoy good general health. However, the burden of psychological problems was significantly higher, since one out of three Greek adolescents reported that their recent psychological state was not good. Girls and older adolescents reported poorer general and psychological health, while adolescents with divorced or separated parents reported worse psychological health only. We studied a number of socioeconomic status variables, but we found robust associations only for the subjective assessment of the presence of financial difficulties in the family and the adolescents' own assessment of their academic performance in school. These two variables were significantly associated with both general and psychological health status, but assessment of academic performance was associated with psychological health in a more linear way. The more objective socioeconomic status variables of parental education and employment/occupational status were not associated with health status in the fully adjusted model, but father's unemployment showed a non-significant trend for an association with worse psychological health in girls only.

### Comparison with other studies

The prevalence of poor self-reported general health is in concordance with evidence from previous studies [8,10]. The most impressive finding though is the significant burden of psychological ill health of Greek adolescents. This finding is in concordance with the results of the KIDSCREEN project. The KIDSCREEN European project took place between 2001 and 2004 in 13 European countries and its aim was to develop a new indicator and to measure well-being and mental health problems in children and adolescents. Greece was one of the countries with the lowest mean scores of positive mental health [27]. A large number of previous studies have used conventional measures of health, such as mortality or morbidity from physical illness, neglecting mental health. Nevertheless, almost every time mental health parameters are investigated it emerges that psychological morbidity is quite prevalent even during adolescence [38]. Studies which focused in the school context resulted in the revelation of "an epidemic" [39]. Moreover, even when alternative measures, such as self-reported health, are applied, the measured quantity is health in general, without discriminating between physical and psychological health. However, when people are asked to rate their health, they tend to assess the status of their physical health [21].

On the other hand, adolescents enjoy good physical health on the whole and many researchers argue that the image of "social equalization in youth" may be the result of the limited variation in the variables chosen [40]. In our study some of the socioeconomic associations we found with worse health were stronger for self-reported psychological health than for self-reported general health (e.g. female gender, assessment of school performance). However, results did not differ substantially for both measures of health status.

Regarding socio-demographic determinants, evidence shows that female gender and non-intact family structure may serve as risk factors for adolescent health [41]. In our study socio-demographic inequalities were also confirmed, since girls, older adolescents and adolescents with divorced parents were more likely to report worse psychological health.

Our findings also show that socioeconomic inequalities in health status are more likely to be reported when subjective socioeconomic indicators are used. The traditional socioeconomic variables of parental education and employment were not associated with health status. Similar findings have been reported by previous studies [11,13,14]. However, unlike previous literature with similar findings, we cannot argue that there are no gradients in adolescent health. The additional use of subjective socioeconomic measures demonstrates significant inequalities and does not allow us to support the argument of social equalization in adolescent health. In this point it is important to note that our data refer to adolescents attending Senior High Schools, which means that socioeconomic status is expected to be more equal among the sample than it is in the general population.

Among traditional socioeconomic measures father's unemployment showed a non-significant trend for an association with worse psychological health in girls only. We could argue that the lack of statistical significance might be due to type II error, which may be caused by the relative small size of the sample of girls with unemployed fathers (n = 27). A possible association between parental unemployment and poor health has been reported by previous studies. Financial stress is considered to be the most important consequence of unemployment, with regard to the health of the unemployed individual or the family members. More specifically, income seems to affect psychological health indirectly via subjectively appraised financial strain [42].

As regards alternative socioeconomic measures, adolescents' subjective view on the financial difficulties of the family, as well as their school performance, were both strongly associated with self-reported general and psychological health. The question about financial difficulties is a frequently used indicator among studies about adult health [16,43] but an uncommon one among studies about adolescents. However, studies which applied this measure demonstrated its significant relationship with adolescents' mental health [8]. A number of studies, which applied similar measures concerning adolescents' subjective perception of the family's financial situation, have also demonstrated a strong association with adolescent health [36,44]. On the other hand, a study which investigated socioeconomic health inequalities using subjective socioeconomic measures ("Family Affluence Scale") in a sample of Greek adolescents did not show any significant correlation with mental health problems [27]. Moreover, studies that used adolescents' position in school as a socioeconomic indicator showed independent correlations with adolescent health [40]. Similar correlations emerged when other subjective measures of "individualistic" socioeconomic status were applied [45].

### Limitations of the study

Limitations of the present study include the cross-sectional nature of our data, which means that the cause and effect relationships cannot be investigated. In addition, health evaluation was based on adolescents' self-reports without parental reports or medical verification. It has been argued that when self-report is used to collect data it may lead in overestimation of the morbidity of the population [46]. Self-reported health though is a widely used indicator with good sensitivity and specificity [20]. Our findings are consistent with evidence not only from studies, where data collection was based exclusively on adolescent' self-report [47], but also from studies which used additionally parental reports [48]. Furthermore, it seems that school-based studies tend to underestimate rather than overestimate morbidity since children suffering from limiting longstanding physical or mental illness may stay at home and not attend schools.

Moreover, psychological health was crudely assessed with a single question that was designed to be very similar to the question on self reported general health. We did so because our aim was to investigate how these questions compare with each other as regards the sociodemographic and socio-economic associations. The simple question on psychological health was highly correlated with the GHQ-12, a commonly used instrument to assess psychological morbidity in surveys around the world. It was also significantly correlated with the emotional sub-scale of the SDQ. According to the results of this study, this question also seems to be a useful indicator of mental health in adolescence capable of demonstrating socioeconomic inequalities in mental health in this period of life. However, as this is a crude assessment, a degree of misclassification is inevitable but most likely this will not lead to systematic errors.

Employment status was also based on adolescents' self-report and this may increase the risk for misclassification. This misclassification is expected to be random and therefore, if it influences the results, this will be towards the null value, i.e. against our hypotheses. Moreover, the question about parental employment status did not include information about the exact occupation and as a result an official "occupational status" classification was not possible. Finally, it should be noted that these results refer to a sample of adolescents attending general upper secondary schools in Greece and generalizations beyond this school setting are not recommended. Furthermore, the sample of the study included only senior high schools. Since there are big differences in perceived health between senior high schools and technical vocational schools, there might also have been differences in the associations between self-rated health and socioeconomic indicators.

## Conclusions

We investigated the issue of socioeconomic health inequalities in a sample of Greek adolescents. It emerges that many adolescents do not enjoy good general health. The burden of psychological problems appears to be significantly higher. When subjective socioeconomic indicators are used, such as subjective assessment of the presence of financial difficulties in the family and adolescent's assessment of their school performance, socioeconomic health inequalities are reported in this period of life too.

Our findings indicate that adolescents' view on the position of their family in the social context, as well as on their own position in the area where they are mainly active, i.e. the school, are more useful in predicting adolescents' health than are parental education or occupation. The relationship between social class and health may be mediated through people's actual experience of their financial situation, affluence and well-being. Traditional socioeconomic measures were designed to express these experiences according to social realities that may do no longer exist. Today societies undergo some major changes. Occupation seems to be unable to guarantee safety and affluence any more. In order to really find what lies beneath, we possibly have to implement additionally some alternative measures, especially when investigating a generation, which is being brought up in the middle of a financial crisis.

## Competing interests

The authors declare no competing interests in relation to this paper.

## Authors' contributions

KM helped in data collection, contributed to the statistical analyses and drafted the manuscript. PS was responsible for the conception and design of the study, helped in data collection, contributed to the statistical analysis and helped in the writing of the paper and interpretation of the results. DN made critical comments and helped in the writing and interpretation of the results.

SB helped in data collection, in the statistical analysis, in the writing of the manuscript and interpretation of the results. AZ made critical comments that helped in the interpretation of the results and the final writing of the paper.

SS made critical comments that helped in the interpretation of the results and the final writing of the paper. VM helped in obtaining funding for the study, in the writing of the paper and interpretation of the results. All authors read and approved the final manuscript.
